# “In ChatGPT-Powered Virtual Influencers We (Dis)Trust?”: The Privacy Paradox and the Double-Edged Sword of Ubiquitous Large Language Model (LLM) Generative AI as a General Purpose Technology (GPT) in a Human-Centered AI Ecosystem

**DOI:** 10.3390/bs16050651

**Published:** 2026-04-26

**Authors:** Seunga Venus Jin

**Affiliations:** Artificial Intelligence and Media Lab (AIM-Lab), Communication Program, Northwestern University in Qatar (NU-Q), Education City, Doha P.O. Box 34102, Qatar; venus@northwestern.edu

**Keywords:** Large language models (LLMs), ChatGPT-powered virtual influencers, AI divide, AI equality, AI-powered digital transformation, need to belong, privacy concerns, ubiquitous AI, general purpose technology (GPT)

## Abstract

“Can ChatGPT become a *general purpose technology*?” “How does the “*privacy paradox*” play a role in adopting ubiquitous AI technologies in a humane AI ecosystem?” To answer these research questions, this study examined the roles of AI equality, trust in the large language model (LLM) ChatGPT, the need to belong, perceived benefits of ubiquitous AI, and privacy concerns about potentially ubiquitous generative artificial intelligence (GenAI) in a human-centered AI ecosystem. Drawing from the emerging literature on the AI divide (vs. AI equality) and AI-powered digital transformation, cross-sectional survey data were collected from current ChatGPT users. The results of testing PROCESS macro models with 5000 bootstrap samples showed the relationship between AI equality and purchase intention is mediated by trust in ChatGPT and is moderated by the need to belong. Privacy concerns about ChatGPT moderate the relationship between AI equality and perceived benefits of ubiquitous GenAI, which, in turn, mediates the relationship between AI equality and purchase intention. Ethical dilemmas in developing an equitable AI ecosystem, practical implications of the “*privacy paradox*” for designing trustworthy and ubiquitous AI interfaces in the dynamically evolving AI-powered digital transformation landscape and electronic marketplaces, and theoretical implications of the ChatGPT epidemic in a humane AI ecosystem for the literature on general purpose technology (GPT) are discussed.

## 1. Introduction

Large language models (LLMs) have garnered widespread public attention, had a societal impact, and led to exponential adoption and rapid integration into various sectors, as evidenced by examples like ChatGPT, which stands for Chat Generative Pre-Trained Transformer (Cahalane & Kirshner, 2025). In the e-commerce domain, LLMs (1) involve search engine integration, synthesizing search results from multiple sources; (2) take the forms of code-writing assistants, enabling developers to build and optimize e-commerce platforms; and (3) take the forms of agents and chatbots, negotiating with other companies’ chatbots to get better prices on products in a transaction (Stokel-Walker & Van Noorden, 2023). An increasing body of research has documented the effectiveness and efficiency of LLM-based product classification, search systems, and recommendation systems in e-commerce environments (Roumeliotis et al., 2024; Wang & Na, 2023).

AI-driven digital transformation has given rise to generative artificial intelligence (GenAI) like ChatGPT, Claude, and Copilot (Javaid et al., 2023); AI-based influencers like ChatGPT-generated virtual influencers (Yu et al., 2024); and AI-powered universes like metaverses (Jin, 2024). ChatGPT is “an AI-powered natural language processing (NLP) tool that comprehends and produces text in response to given commands” (Javaid et al., 2023, p. 127). ChatGPT-4o is even “omnimodal” in that it can generate any kind of data including text, image, audio, and video (Growcoot, 2025). The integration of GPT-4o, designed to process and generate various data forms, multiple modalities of media, and a multitude of data, marks a pivotal advancement in ChatGPT’s capabilities (Ghacks.Net, 2025). The latest generation of the GPT models from OpenAI like ChatGPT-5, ChatGPT-5.2, and ChatGPT-5.3-Codex-Spark also supports and extends multiple formats and even handles more complex input types. Thus, ChatGPT has the capability to generate textual, pictorial, audio, and video content, allowing for the creation of immersive and engaging content for AI-powered virtual influencers (Yu et al., 2024).

Virtual influencers refer to computer-generated images (CGIs) or animated digital characters that are designed and managed by organizations to connect with consumers and influence their purchasing decisions and behaviors (Bringé, 2022). AI-powered virtual influencers are “generated through a fusion of 3D modeling and AI to react to contextual situations and stimuli” (Liyanaarachchi et al., 2024, p. 3). From marketing perspectives, AI-powered virtual influencers have the potential to provide brands with (1) enhanced control and safety over their marketing campaigns that align with their brand identity and desired brand image, thus ultimately protecting their brand reputation and (2) practical tools to encourage consumer engagement (Jin & Viswanathan, 2024; Liyanaarachchi et al., 2024; Yu et al., 2024).

The present research aims to examine the roles of AI equality (versus AI divide) (Bircan & Özbilgin, 2025), the need to belong (Baumeister, 2012), and the dynamics of the “privacy paradox” (Barth & de Jong, 2017) in affecting consumers’ behavioral intention to purchase products promoted by ChatGPT-powered virtual influencers in the underexplored domain of Human–AI Interaction (HAII) in AI-driven digital transformation. The central research question (RQ) of the present study is as follows: “What are predictors of consumers’ behavioral intentions to purchase products promoted by ChatGPT-powered virtual influencers?” To answer this exploratory question, an empirical study is drawn from the emerging literature on AI equality in a humane AI ecosystem (Jin, 2024; Jin & Ryu, 2025; Skare et al., 2024) and AI-powered virtual influencers (Jin, 2023; Jin & Viswanathan, 2024; Liyanaarachchi et al., 2024), as further elaborated in the following sections.

## 2. Conceptual Frameworks and Hypotheses

### 2.1. AI Equality (vs. AI Divide)

Third-level digital equality, referring to “the ability to exploit benefits in a digital-driven market to improve one’s life chances” (Ragnedda, 2017, p. 5), is correlated with “the differential offline outcomes that people obtain from their use of digital technologies” (Calderón Gómez, 2021, p. 2536). Drawing from the third-level digital equality literature (Jin, 2024; Ragnedda, 2017; Van Deursen & Helsper, 2015) and applying the conceptual definition of third-level digital equality to AI-powered digital transformation, “third-level AI equality” can be operationally defined as “*the equality regarding AI users’ capacity to translate their AI access and usage into favorable offline outcomes*.” Conceptually, AI equality can be an antonym to AI divide.

A report by the International Monetary Fund (IMF) indicates significant disparities in AI preparedness and readiness among nations, highlighting a growing divide between those equipped to leverage AI and those at risk of falling behind (Cazzaniga et al., 2024). The AI divide refers to “a chasm between those who can fully engage with and benefit from AI technologies and those who are left on the periphery” (Bircan & Özbilgin, 2025, p. 5). The AI divide is not only a technical issue but also a sociological one, which is deepened with the division between “technophile and technophobe” segments of society (Archer, 2021; Bircan & Özbilgin, 2025; Jin, 2024; Youn & Jin, 2021). Thus, the AI divide can be examined between nations at the global level or within a country at the local level. The current research particularly focuses on perceived AI equality (vs. divide) between people within a particular nation/society (i.e., USA) at the local level.

Prior research shows that people with higher digital access (first-level digital equality) (van Dijk, 2006) and usage (second-level digital equality) (Hargittai, 2002) show more trust in AI-powered digital transformation since they believe they can further benefit from access and usage of emerging AI technologies (third-level digital equality) (Jin, 2024). Research indicates a positive and statistically significant correlation among AI technology adoption, AI capital stock accumulation, and wealth disparity (Skare et al., 2024), which empirically demonstrates the impact of third-level AI equality on real-life benefits and outcomes. Thus, third-level AI equality is an important factor to consider in constructing a humane, equitable, and socially sustainable AI ecosystem, whereas the AI divide may be an obstacle to building an equitable AI ecosystem both at the local level and at the global level.

### 2.2. “In ChatGPT We Trust?”: The Mediating Role of Trust

Trust is also an integral component of constructing a socially responsible and equitable AI ecosystem. GenAI has the potential and capacity to enhance consumer experience and merchant productivity through AI-generated product description, product review sentiment analysis, efficient and accurate product classification (Ghaffari et al., 2024), interactive chatbots, human-like virtual assistants, language translation services, content summarization tools, and so forth (Fanni et al., 2023) in AI-driven digital transformation. Empirical research shows that third-level digital equality is a positive predictor of consumers’ trust in AI-empowered digital transformation, and trust is a positive predictor of behavioral intention to adopt AI-empowered interfaces for virtual commerce (Jin, 2024). Therefore, it can be reasonably hypothesized that the more users can translate AI access and usage into favorable real-life outcomes, the more they can trust ChatGPT (H1); and the more consumers trust ChatGPT, the more they are willing to purchase products promoted by ChatGPT-powered virtual influencers (H2) in AI-based virtual commerce.

**Figure 1:**
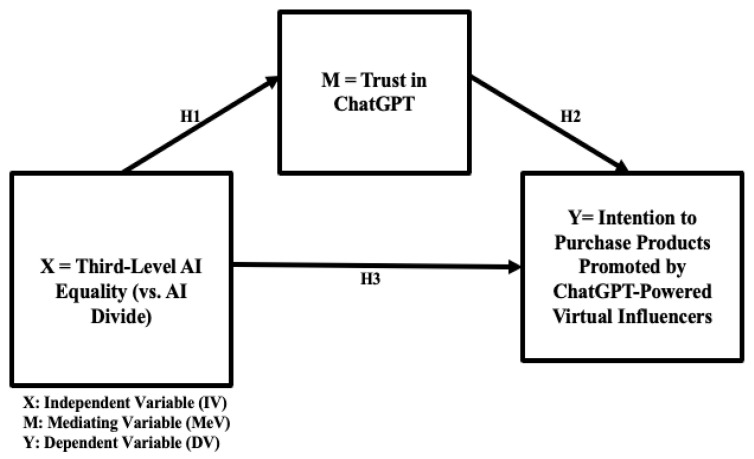
Conceptual model 1: the mediating role of trust in ChatGPT (H1, H2, and H3).

Building upon its transformative potential to unlock the power of language, NLP fosters “the creation of intelligent systems capable of understanding and communicating with humans in a manner that feels natural and intuitive” (Roumeliotis et al., 2024, p. 3). ChatGPT-powered virtual influencers, as anthropomorphic agents that use human language and manifest human-like attributes, may play an increasingly important role in Human–AI Interaction (HAII) in the emerging context of AI-driven e-commerce. Empirical research on LLM-enhanced product recommendation reports that LLMs for personalized user embeddings improved consumers’ click-through rate (CTR) and purchase rate in e-commerce (Katlariwala & Gupta, 2024). A robust understanding of users’ trust in conversational artificial intelligence (CAI) is crucial for humane, customer-centric AI user interface (UI) development and user experience (UX) (Leschanowsky et al., 2024). Empirical studies show that trust is a significant mediator between the third-level digital equality and behavioral intention to adopt AI-powered interfaces for virtual commerce (Jin, 2024) and AI-powered metaverses (Jin & Ryu, 2025). Trust plays a significant mediating role in consumers’ adoption of a wide range of innovative and emerging technologies (Amoako et al., 2021; Koroma et al., 2022; Lu et al., 2022). For example, in the context of AI-powered smart technology adoption and smart cities for equitable societies, Bittencourt et al. (2024) and Bittencourt et al. (2025) emphasized the importance of trust in fostering citizen engagement. Also, research demonstrates that greater levels of trust encourage greater use of e-government, while inequality in the access and possible use of e-government is a reason for concerns and challenges in adopting innovations (Pérez-Morote et al., 2020). Therefore, it can be reasonably hypothesized that trust in ChatGPT is a mediator between third-level AI equality and behavioral intention to purchase products recommended by ChatGPT-powered virtual influencers (H3) in AI-based virtual commerce. The proposed mediation model is visually presented in Figure 1.

**H1.**  *Third-level AI equality (vs. AI divide) is a positive predictor of users’ trust (vs. mistrust) in ChatGPT.*

**H2.**  *Trust in ChatGPT is a positive predictor of ChatGPT users’ intention to purchase products promoted by ChatGPT-powered virtual influencers.*

**H3.**  *Trust in ChatGPT mediates the positive relationship between higher third-level AI equality (lower AI divide) and ChatGPT users’ higher intention to purchase products promoted by ChatGPT-powered virtual influencers.*

### 2.3. AI Divide (vs. AI Equality) and a Sense of Belonging: The Moderating Role of the Need to Belong

**Figure 2:**
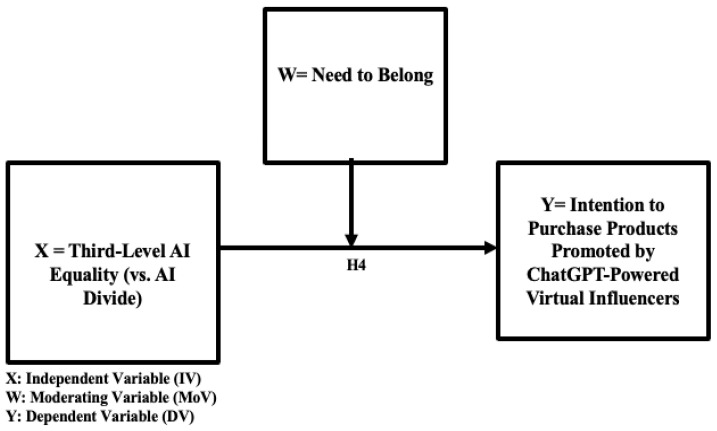
Conceptual model 2: the moderating role of the need to belong (H4).

The need to belong refers to human motivation to form and sustain social connections (Baumeister, 2012). Prior research shows that (1) social media users’ loneliness moderates the relationship between the humanness (versus eeriness) of AI-powered virtual influencers and empathy and compliance with them in AI-driven digital transformation (Jin, 2023), (2) people with a higher need to belong follow virtual influencers more (Jin & Viswanathan, 2024), and (3) heavy ChatGPT users feel lonelier (Mitchell, 2025; Phang et al., 2025). More specifically, Jin (2023) empirically confirmed the moderating effect of consumers’ loneliness on the relationship between the humanness (vs. eeriness) of virtual influencers and consumers’ empathy with them. When the perceived eeriness of virtual influencers was high, lonely consumers indicated greater empathy with virtual influencers than non-lonely consumers. In contrast, when the perceived humanness of virtual influencers was high, consumers indicated an equivalently high level of empathy with virtual influencers regardless of their loneliness level (Jin, 2023). This finding suggests the importance of examining consumers’ psychological state in Human–AI Interaction (HAII). Similarly, Jin and Viswanathan (2024) empirically showed that the need to belong moderates the relationship between consumers’ following status (following versus non-following of AI-powered virtual influencers) and behavioral intention in AI-driven digital marketing. For those consumers with a lower need to belong, the following status had no effect on the perception of AI-powered virtual influencers’ personalization benefit. On the contrary, for those lonely consumers with a higher need to belong, followers’ perception of AI virtual influencers’ personalization benefits was higher than non-followers. With regard to the association between ChatGPT usage pattern and emotional well-being, users who self-reported as being lonelier exhibited more affective cues in conversation with the ChatGPT model (Phang et al., 2025). Similarly, users who self-reported as being less social tended to have more affective cues in conversation with the ChatGPT model (Phang et al., 2025). Socially vulnerable populations with higher social exclusion tended to encounter more difficulties in using digital technologies and gaining benefits from them, thus leading to further inequalities (Ragnedda et al., 2022). Based on these theoretical rationales and empirical findings from previous studies, the current research attempts to test the moderating effect of consumers’ need to belong on the relationship between AI equality and their behavioral intention to purchase products promoted by ChatGPT-powered virtual influencers (H4). No prior research has addressed the two-way interaction effects of consumers’ need to belong (psychological state and well-being dimension) and AI equality (infrastructure and resource dimension) on behavioral intention to purchase products promoted by ChatGPT-powered virtual influencers in Human–AI Interaction (HAII) in AI-driven e-commerce environments. The proposed moderation model is visually presented in Figure 2.

**H4.**  *The need to belong moderates the positive relationship between higher third-level AI equality (lower AI divide) and ChatGPT users’ higher intention to purchase products recommended by ChatGPT-powered virtual influencers.*

### 2.4. “Can ChatGPT Become a GPT?”

A general purpose technology (GPT) is a term coined to describe “technology characterized by the potential for pervasive use in a wide range of sectors and by their technological dynamism” (Bresnahan & Trajtenberg, 1995, p. 84), “a new method of producing and inventing that is important enough to have a protracted aggregate impact” (Jovanovic & Rousseau, 2005, p. 1181), and “new tools that are powerful enough to accelerate overall economic growth and transform economies and societies” (Stackpole, 2024). Thus, three characteristics of GPTs include: (1) pervasiveness and widespread use; (2) potential for improvement and innovation; and (3) innovation spawning in a wide variety of application industries (Bresnahan & Trajtenberg, 1995; Goldfarb et al., 2023). GenAI has the potential to be the next invention to join the GPT category (Stackpole, 2024) given that GenAI is a game-changing technology in the rapidly evolving digital transformation landscape across a multitude of sectors and domains, by spurring societal change at an exponential rate due to its accessibility and ease of diffusion (McAfee, 2024). The present research examines the double-edged sword of ChatGPT as a potential general purpose technology (GPT), by testing moderated mediation effects of privacy concerns and perceived benefits of ubiquitous ChatGPT as elaborated below.

### 2.5. “Double-Edged Sword” and “Privacy Paradox”: The Moderated Mediation of Ubiquitous AI and Privacy Concerns

ChatGPT has significantly increased societal awareness of LLMs and has been rapidly and impactfully integrated into a broad spectrum of sectors (Cahalane & Kirshner, 2025), thus demonstrating its potential to become a general purpose technology (GPT) (Stokel-Walker & Van Noorden, 2023). One of the dark sides of the ChatGPT epidemic and LLMs is privacy concerns. A study examining privacy risks of using general purpose LLMs found that these models could leak private and sensitive information to adversaries (X. Pan et al., 2020). LLMs have grown exponentially and have utilized unprecedentedly large datasets of natural language, thus making privacy risks in LLMs a significant problem (Brown et al., 2022).

However, while many users show theoretical interests in their privacy, this rarely translates into actual privacy-protective behavior (Joinson et al., 2010). Furthermore, even when users are aware of the risks and highly concerned about a potential breach of privacy, users willingly disclose large amounts of sensitive personal data (Hallam & Zanella, 2017). The “privacy paradox” refers to this incongruity between privacy concerns (perception) and online self-disclosure activities (behavior) (Barth & de Jong, 2017), thus depicting the discrepancy between consumers’ attitudes and behaviors in private information disclosure (Barnes, 2006).

Consumers engaged with virtual influencers may be “a highly vulnerable segment to data breaches with the increased disclosure of personal information, IP addresses, and social media profiles” (Liyanaarachchi et al., 2024, p. 4). Furthermore, the celebrity status and increasing social influence of virtual influencers (Kim & Park, 2024) may exacerbate the privacy paradox since virtual influencers’ status, fame, and persona may overshadow consumers’ ability to discern and determine the severity of privacy risks (Liyanaarachchi et al., 2024). Therefore, the privacy paradox in relation to consumers’ interaction with AI-powered virtual influencers warrants theory-driven hypothesis testing and empirical examination from both scholarly and managerial perspectives.

**Figure 3:**
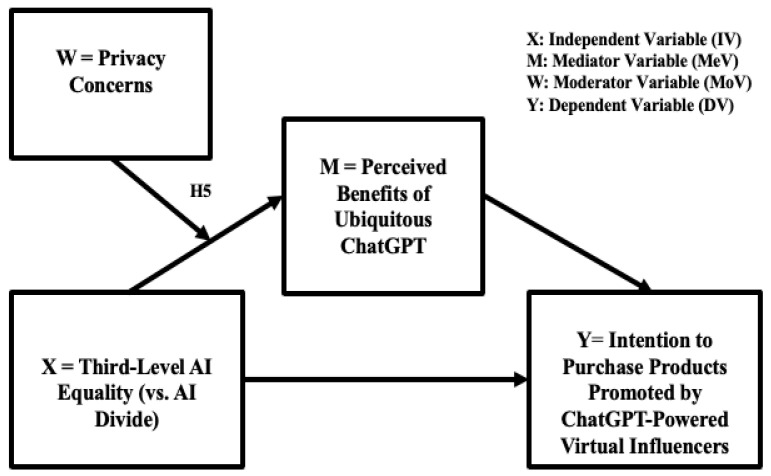
Conceptual model 3: the moderated mediation effects of ubiquitous AI and privacy concerns (H5).

While the privacy paradox makes it challenging for companies to convince consumers to interact with AI-enabled systems (Lalicic & Weismayer, 2021; Ostrom et al., 2019), consumers may still adopt AI technologies when those technologies become ubiquitous and when consumers perceive the benefits of using ubiquitous AI technologies to outweigh privacy concerns. The tension between consumers’ perceived benefits of ubiquitous ChatGPT as a potential general purpose technology (GPT) and consumers’ privacy concerns adds another dimension to the privacy paradox in the dynamic process of AI-driven digital transformation. An experimental study empirically showed that privacy and trust at a situational level interact such that high trust compensates for low privacy protection, and vice versa (Joinson et al., 2010). The present research attempts to elucidate the psychological mechanism behind the privacy paradox in AI-powered virtual commerce by testing the moderating role of privacy concerns in explaining the mediating role of consumers’ perceived benefits of ubiquitous AI technologies. The proposed moderated mediation model is visually presented in Figure 3.

**H5.**  *The mediating effect of users’ perceived benefits of ubiquitous ChatGPT on the positive relationship between higher third-level AI equality (lower AI divide) and users’ higher intention to purchase products recommended by ChatGPT-powered virtual influencers is moderated by users’ privacy concerns about ChatGPT.*

The proposed conceptual models with the relevant hypotheses are visually presented in Figure 1, Figure 2 and Figure 3 (H1, H2, and H3 in Figure 1; H4 in Figure 2; and H5 in Figure 3).

## 3. Methods

### 3.1. Data Collection and Participants

Cross-sectional survey data were collected from ChatGPT (GPT-4) users in the United States (N = 467; gender composition: 56.3% males and 43.5% females; education level: 0.4% completed some high school, 4.3% high school graduate, 2.1% completed some college, 82.5% bachelor’s degree, 10.7% master’s degree or higher; ethnic background: 91.2% White/Caucasian, 0.6% Black/African American, 0.8% Hispanic/Latin American, 5.3% Asian or Asian Indian, 1.0% American Indian or Alaska Native, 0.4% Mixed race, 0.2% Other, 0.4% prefer not to answer) using the CloudResearch Prime Panel. Empirical research indicates that Prime Panel participants, compared to MTurk workers, are more diverse in age, family composition, religiosity, education, and political attitudes, thus demonstrating improved representativeness and higher data quality (Chandler et al., 2019). The survey questionnaire prepared on the Qualtrics platform was embedded in the CloudResearch data collection tool. Informed consent was obtained from all human subjects involved in the study in accordance with the ethical approval from the IRB review. All participants electronically signed the informed consent form before filling out the survey questionnaire.

### 3.2. Survey Instruments and Measures

The survey questionnaire included clear definitions and explanations about the following terminologies. “Artificial Intelligence (AI) is defined as “the capability of a machine to imitate intelligent human behavior”. Artificial Intelligence (AI) is the simulation of human intelligence processes by machines, especially computer systems. Artificial Intelligence (AI) examples include chatbots like ChatGPT, smart speakers/smart assistants, manufacturing robots, self-driving cars, virtual travel booking agents, and social media algorithms.” “ChatGPT stands for “Chat Generative Pre-Trained Transformer”. ChatGPT is a large language model-based chatbot developed by OpenAI and launched on 30 November 2022. ChatGPT is notable for enabling users to refine and steer a conversation towards a desired length, format, style, level of detail, and language used.” “ChatGPT-powered virtual influencers can (1) engage in conversations and participate in real-time interactions with their audience and (2) gather information about their followers, learn their preferences, and deliver targeted and personalized content.”

All the measures were borrowed from previously validated scales and measurement items. Third-level AI equality, as the exogenous variable, was measured with a modified version of the economic commerce outcome dimension of the third-level digital equality scale (Van Deursen & Helsper, 2015). Only the economic commerce outcome dimension of the third-level digital equality scale (Van Deursen & Helsper, 2015) was used given that the main dependent variable of this study was “purchase intention”. Trust in ChatGPT was measured with a modified version of the consumer trust scale (Y. Pan & Zinkhan, 2006). The need to belong was measured with the need to belong scale (Leary et al., 2013). Perceived ubiquitous benefits of ChatGPT were measured with ubiquitous AI benefit items (Lalicic & Weismayer, 2021; Xu et al., 2011). Privacy concerns about ChatGPT were measured with privacy concern items from previous research on AI-enabled travel service agents (Lalicic & Weismayer, 2021) and smartphone-embedded tracking (Ketelaar & van Balen, 2018). Behavioral intention to purchase products promoted by ChatGPT-powered virtual influencers was measured with a modified version of the buying intention scale (Holzwarth et al., 2006). The list of variables, the results of reliability testing, and sample measurement items are presented in Table 1.

### 3.3. Data Analysis

To answer the exploratory research question (RQ), data were analyzed using a neural network analysis with a gradient descent optimization algorithm, by entering AI equality, trust in ChatGPT, the need to belong, perceived benefits of ubiquitous ChatGPT, and privacy concerns as the input layers, and purchase intention as the output layer. The ratio of training data to testing data was set at 70% to 30%. To test the theory-driven formal hypotheses (H1, H2, H3, H4, and H5), mediation and moderation analyses were conducted using PROCESS SPSS Version 5.0 Beta 2.1 Macro (Hayes, 2022).

## 4. Results

### 4.1. Descriptive Statistics and Bi-Variate Correlation

The results of descriptive statistics and a bi-variate Pearson correlation matrix are presented in Table 2.

### 4.2. The Mediating Role of Trust in ChatGPT (H1, H2, and H3)

**Figure 4:**
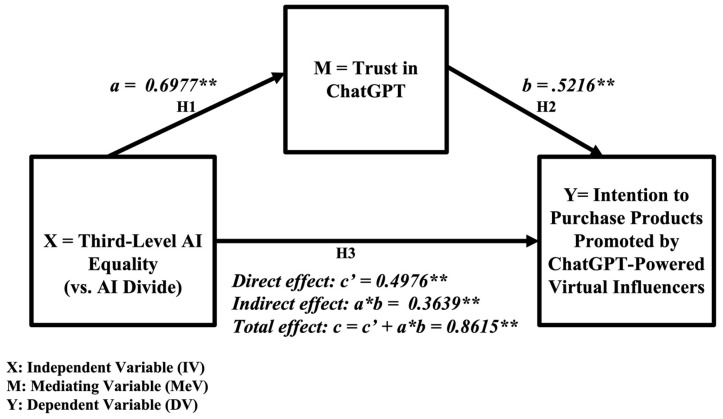
Empirical results: the mediating role of trust in ChatGPT (H1, H2, and H3). ** *p* < 0.01.

Data were analyzed using PROCESS SPSS Version 5.0 Beta 2.1 Macro with 5000 bootstrap samples (Hayes, 2022). PROCESS Model 4 was used to test the mediating effect of trust in ChatGPT. Third-level AI equality (vs. AI divide) was a positive (vs. negative) predictor of trust in ChatGPT (b = 0.6977; t = 23.7390; *p* = 0.000), thus supporting H1. Trust in ChatGPT was a positive predictor of behavioral intention to purchase products recommended by ChatGPT-powered virtual influencers (b = 0.5216; t = 13.5185; *p* = 0.000), thus supporting H2. The results revealed a significant indirect effect of trust in ChatGPT on behavioral intention to buy products recommended by ChatGPT-powered virtual influencers (b = 0.3639; Lower Limit Confidence Interval [BootLLCI] = 0.2027; Upper Limit Confidence Interval [BootULCI] = 0.5292), thus supporting H3. Furthermore, the direct effect of AI equality (vs. AI divide) on behavioral intention in the presence of the mediator (trust in ChatGPT) was also found to be significant. Hence, trust in ChatGPT partially mediated the relationship between AI equality (vs. AI divide) and purchase intention. The results of mediation analysis are presented in Figure 4, and the mediation analysis summary is presented in Table 3 (top) (PROCESS Model 4).

### 4.3. The Moderating Role of the Need to Belong (H4)

**Figure 5:**
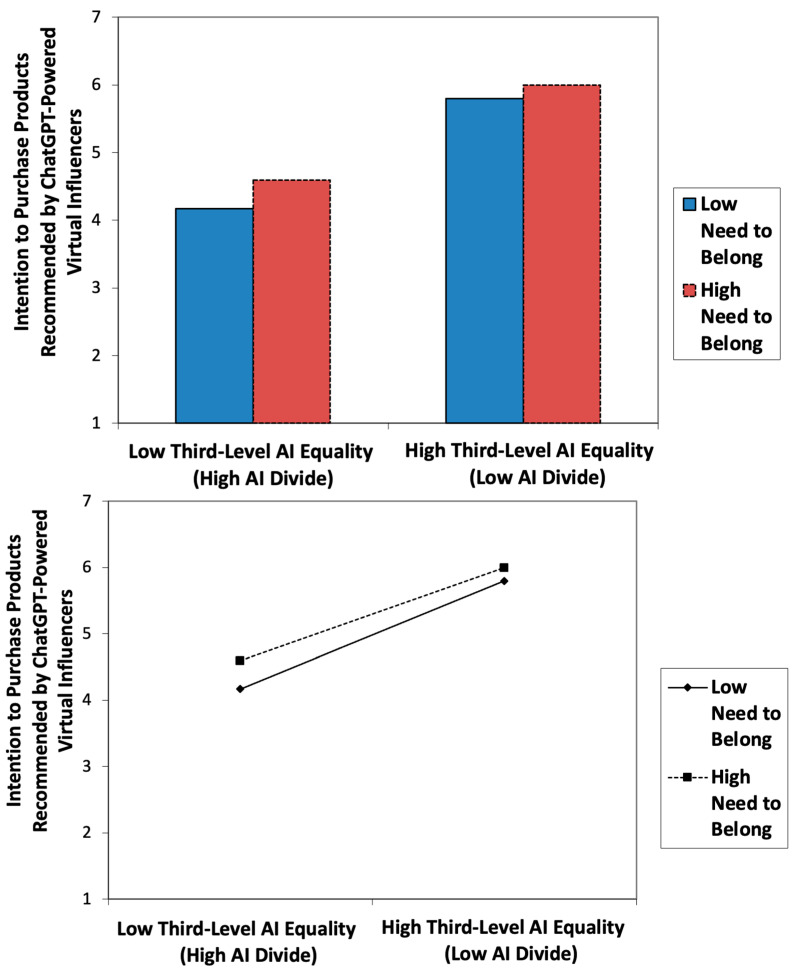
Empirical results: the moderating role of the need to belong (H4).

PROCESS Model 1 was used to test the moderating effect of the need to belong, as a continuous variable, on the relationship between AI equality (vs. AI divide) and behavioral intention to purchase products recommended by ChatGPT-powered virtual influencers. The results revealed a significant moderating effect of the need to belong (b = −0.0561; t = −3.2711; *p* = 0.0012), supporting H4. The results of slope analysis to further understand the nature of the moderating effects are graphically presented in Figure 5. As shown in Figure 5 (bottom), the line is steeper for a lower need to belong, which shows that at lower levels of a need to belong, the impact of AI equality (vs. AI divide) is stronger in comparison to a higher need to belong. Furthermore, as the level of AI equality decreases (AI divide increases), the strength of the relationship between the need to belong and behavioral intention to purchase products recommended by ChatGPT-powered virtual influencers increases.

### 4.4. The Moderated Mediation Effects of Privacy Concerns and Ubiquitous Benefits (H5)

**Figure 6:**
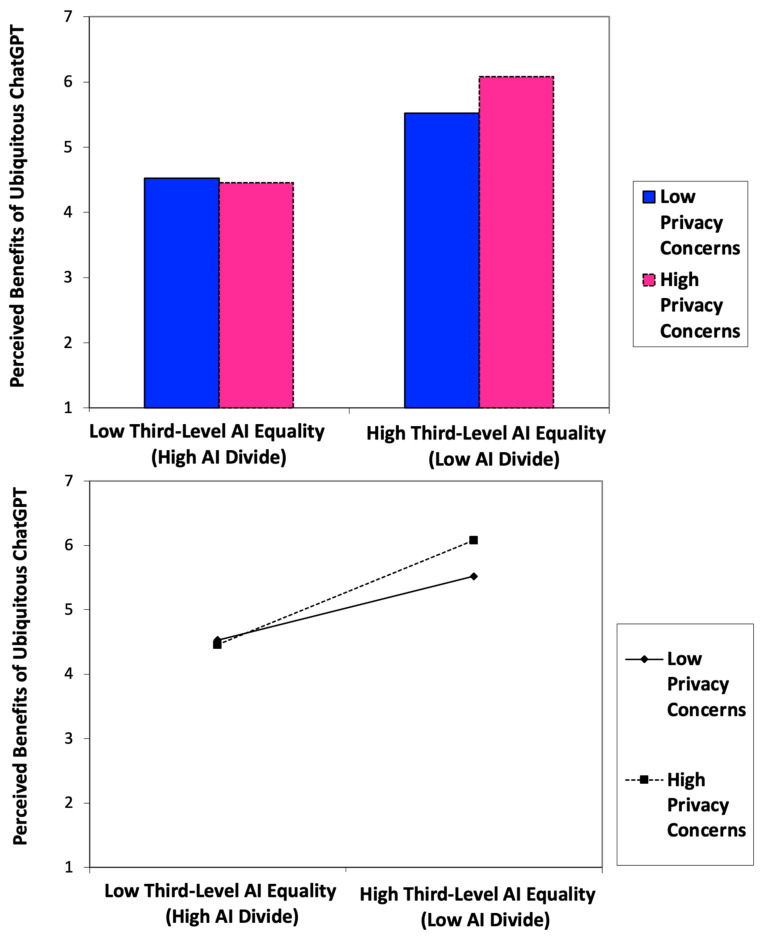
Empirical results: the moderated mediation effects of ubiquitous AI and privacy concerns (H5).

A moderated mediation model, with privacy concerns about ChatGPT as the moderator and perceived benefits of ubiquitous ChatGPT as the mediator, was tested using PROCESS SPSS Version 5.0 Beta 2.1 Macro Model 7. The index of moderated mediation (index = 0.0570; 95% confidence interval = BootLLCI [lower] = 0.0101; BootULCI [upper] = 0.1071) is significant, thus supporting **H5**. The results of the moderated mediation analysis are presented in Table 3 (bottom) (PROCESS Model 7) and the two-way interaction effects between AI equality (vs. AI divide) and privacy concerns on perceived benefits of ubiquitous ChatGPT are graphically presented in Figure 6. For those with low AI equality (high AI divide), privacy concerns had no effect on perceived benefits of ubiquitous ChatGPT. In contrast, for those with high AI equality (low AI divide), perceived benefits of ubiquitous ChatGPT were higher for those with high privacy concerns than for those with low privacy concerns.

## 5. Discussion

### 5.1. Key Findings and Theoretical Contributions

AI has the transformative potential to significantly boost productivity and foster economic growth by automating tasks and driving innovation but poses risks of labor market disruption and AI divide (Mandon, 2025) both at the microeconomic and macroeconomic levels (Skare et al., 2024) as well as at the local and the global levels. The current study fills a gap in the emerging literature on the AI divide (Archer, 2021; Bircan & Özbilgin, 2025) by examining relevant mediators and moderators in explaining the relationship between AI equality and persuasion induced by ChatGPT-powered virtual influencers in the underexplored domain of Human–GenAI Interaction in e-commerce environments. More specifically, this research elucidated (1) the significant mediating effect of trust and (2) the significant moderating effect of the need to belong, as psychological state and well-being dimensions, on the relationship between AI equality, as an infrastructure and resource dimension, and consumers’ behavioral intention to purchase products promoted by ChatGPT-powered virtual influencers.

The results of statistical analyses highlight the importance of trust in ChatGPT (Figure 1 and Figure 4) and the two-way interaction between AI equality and the need to belong (Figure 2 and Figure 5) in AI-driven virtual commerce. The results of moderation analysis show that when AI equality is low (high AI divide), consumers with ahigh need to belong indicate a higher purchase intention than those with a low need to belong, consistent with previous theoretical propositions and empirical findings (Jin & Viswanathan, 2024) as well as the emerging literature on the role of loneliness in light of the ChatGPT epidemic (Mitchell, 2025; Phang et al., 2025). In contrast, when AI equality is high (low AI divide), there is no difference between consumers with a high need to belong and those with a low need to belong with regard to purchase intention. This two-way interaction effect of AI equality (vs. AI divide) and consumers’ need to belong demonstrates the importance of examining individual-level consumer psychology (e.g., loneliness, need for belonging, and psychological well-being) when assessing the impact of societal-level infrastructure (e.g., availability of and access to AI resources). Thus, the present study adds timely dialogs, relevant conceptual/theoretical frameworks, and original empirical data to the emerging literature on AI equality (vs. AI divide) (Jin, 2024; Jin & Ryu, 2025), AI-powered virtual influencers in digital marketing (Jin & Viswanathan, 2024), and the roles of consumer psychology and well-being in Human–AI Interaction (HAII) (Jin, 2023). Ultimately, these findings regarding the two-way interaction effects of the AI divide and need to belong on buying intention speak to the ethical dilemma of inviting lonely and vulnerable consumers into the AI ecosystem and persuading them for marketing purposes. In other words, in the boundary condition of low AI equality (high AI divide), those consumers with limited access to the AI ecosystem tend to indicate a stronger desire to benefit from AI-powered virtual commerce and a higher tendency to be persuaded by AI-powered virtual influencers, thus suggesting higher vulnerability of consumers with a higher need to belong but with lower access to AI resources in the emerging marketing domain of persuasive communication by AI virtual influencers. This finding is also consistent with the recent empirical finding that suggests that the benefits of AI capital may not be universally advantageous and could exacerbate wealth disparities (Skare et al., 2024).

Furthermore, the empirical results show that perceived benefits of ubiquitous ChatGPT as a potential general purpose technology (GPT) were higher for those with high privacy concerns than for those with low privacy concerns when AI equality is high (low AI divide) in AI-powered digital transformation, thus further confirming the notion of the “privacy paradox” by adding original empirical evidence of the “privacy paradox” (Barth & de Jong, 2017) to the novel context of ChatGPT-powered virtual influencer marketing and e-commerce. This is an intriguing finding as it suggests that particularly in the boundary condition of high AI equality, consumers with high privacy concerns and high perceived benefits of ubiquitous ChatGPT empirically showed an actual experience of the “privacy paradox” (Figure 3 and Figure 6). In contrast, there was no difference between those with high versus low privacy concerns regarding their perceived benefits of ubiquitous ChatGPT in the boundary condition of a high AI divide. More specifically, under a high AI divide, perceived benefits of ubiquitous ChatGPT were the lowest regardless of the level of privacy concerns (Figure 6). This article has the potential to provoke further theoretical discourses about the psychological, social, and ethical implications of AI-empowered digital transformation by addressing factors to consider in building and sustaining an inclusive, equitable, and socially responsible AI ecosystem, that is, an AI ecosystem that is inclusive of diverse market segments and diverse consumer populations from various socioeconomic backgrounds, providing equitable access to vulnerable populations, and addressing privacy concerns in a socially responsible manner. Ethical dilemmas in developing an equitable AI ecosystem, practical implications of the “privacy paradox” for designing trustworthy and ubiquitous AI interfaces in the dynamically evolving AI-powered digital transformation landscape, and theoretical implications of the ChatGPT epidemic for the literature on general purpose technology (GPT) in electronic marketplaces within a human-centered AI ecosystem need to be further discussed.

To summarize, this study contributes to the literature in three ways. First, it extends the concept of a third-level digital divide (Van Deursen & Helsper, 2015) to the emerging domain of generative AI (GenAI), by introducing the construct of AI equality to the novel domain of AI-powered virtual influencer marketing and e-commerce. Second, it integrates psychological drivers (i.e., need to belong) (Baumeister, 2012) with structural technological factors (AI equality and AI infrastructure) to explain consumer susceptibility to AI-powered persuasion, by empirically testing and verifying the significant moderating effects of consumers’ need to belong. Third, it provides empirical evidence for the privacy paradox (Barth & de Jong, 2017) within the context of AI-driven virtual influencer marketing, by demonstrating the significant moderating effects of privacy concerns in explaining the mediating effects of benefits of ubiquitous ChatGPT technologies as a potential general purpose technology (GPT) on the relationship between third-level AI equality and purchase intentions.

### 5.2. Practical and Managerial Implications

The empirical findings from this study can provide practical insights on the design of ChatGPT-powered virtual influencers and AI agent-based virtual commerce interfaces. First, from the marketer’s perspective, it is crucial to build trust between humanoid virtual influencers and consumers to bring about persuasion, as empirically shown by the meditation effect of trust on the relationship between AI equality and persuasion.

Second, the findings about the moderating effects of consumers’ need to belong and privacy concerns buttress the importance of UI/UX designers’ robust understanding of affective computing, referring to “human-computer collaboration where the user’s emotions can be identified and an appropriate response can be formed in return” (Picard, 1997; Shoumy et al., 2020, p. 2), in the evolving AI-powered digital transformation landscape. For instance, depending on the number of followers/following, which can be measured from consumers’ social media profiles, consumers’ need to belong can be indirectly estimated and these metrics can be utilized to better serve consumers’ social needs in virtual influencer marketing and AI-powered social commerce. Furthermore, depending on the level of privacy concerns, which can be pre-measured by consumers’ privacy settings in e-commerce environments, ChatGPT-powered virtual influencers and their LLM-based marketing messages can be designed and customized differentially to best serve consumers’ various needs and preferences.

Third, UI/UX designers, digital marketers, and brand managers may benefit from the present empirical findings about the moderated mediation effects of ubiquitous AI and privacy concerns when they consider adopting ubiquitous AI agents for AI-driven virtual commerce interfaces in various service industry sectors (e.g., banking/finance, beauty, fashion, education, healthcare, travel/hospitality, gaming/entertainment, social networking/online dating, and more). For example, consumers’ privacy concerns may be more relevant to banking/financial services and healthcare sectors where they actually need to provide private, sensitive, and/or confidential information to AI-enabled chatbots for actual transactional communication and real-life problem solving. The need to belong and loneliness may be more relevant to social networking/online dating industry sectors. Thus, it is crucial for UI/UX designers, digital marketers, and brand managers to have a solid and holistic understanding of their market segments and target consumers’ psychological states, needs, and wants when designing and adopting AI-powered chatbots, virtual influencers, and brand ambassadors for their respective industry/service sectors.

### 5.3. Limitations and Suggestions for Future Research

First, there are a number of methodological limitations that need to be truthfully acknowledged. The use of cross-sectional data limits the current study’s capacity to make claims about causal inferences between the independent variables and the dependent variables. The methodological limitation of self-reported dependent variables weakens the practical validity of the current findings. Relatedly, the limitations with operationalization of general purpose technology (GPT)-related measures need to be addressed in future research since the current research only indirectly operationalized and measured general purpose technology (GPT)-related perception by using measurement items about “ubiquitous benefits” of ChatGPT. Despite the timely and relevant nature of the conceptualization of ChatGPT as a general purpose technology (GPT), these methodological issues with indirect measures need to be addressed to improve the measurement validity of emerging research on these concepts. Follow-up studies need to operationalize, develop scales, and measure general purpose technology-related perception and evaluation more directly rather than relying on existing measures that are only conceptually and theoretically linked to the key characteristics (i.e., ubiquitousness) of general purpose technology. Furthermore, the generalizability of the current empirical findings is limited due to the US-only sample and relatively well-educated people, which hampers making broader claims about global AI ecosystems and generalizability beyond the skewed samples. The study was based on CloudResearch Prime Panel users who are already current ChatGPT users. Although participants were purposefully recruited (i.e., purposive sampling) only from ChatGPT users to ensure relevance of the study sample to the study population, the current sample has innate limitations including possible self-selection bias, possible overestimation of trust in AI systems, and limited cross-cultural generalizability. Although CloudResearch Prime Panel respondents are considered reliable and trustworthy human participants, professional survey respondents may be different from unpaid individuals simply answering a survey. Another methodological consideration for this line of future research is the potential benefit from machine learning and explainable AI algorithms which help provide a detailed analysis of the features affecting the outcome variables (ForouzeshNejad et al., 2025).

Second, the roles of human emotion, AI’s responses to human emotions, trust between humans and AI, human values, and psychological well-being in the emerging domains of HAII and affective computing need to be further examined using a more sophisticated experimental design. For example, different levels of privacy settings (e.g., low–medium–high) as well as different valences (positive vs. negative emotion) and types of emotion-evoking scenarios (e.g., happiness, excitement, enthusiasm, pride, inspiration, sadness, fear, anger, shame, anxiety, and more) in the AI-powered virtual commerce/marketing domain (Pantano & Scarpi, 2022) can be experimentally primed and manipulated. After the experimental manipulation of privacy settings and valences/types of emotions, consumers’ peri-experimental experiences (e.g., emotional connection, parasocial interaction, and feelings of social presence) and post-experimental emotional responses (e.g., feelings of being heard/supported by ChatGPT-powered virtual influencers, frustration, satisfaction/dissatisfaction, envy, surprise, and relief) during and after their interaction with the AI agents can be measured as mediating and endogenous variables other than trust and purchasing intention.

Third, there are other individual difference factors that can be considered and factored into the AI equality (vs. AI divide) equation such as consumers’ AI literacy, referring to “the ability to understand, use, and critically assess AI systems” (Mandon, 2025, p. 3) and “the ability to understand, interact with, and critically evaluate AI systems and AI outputs” (Lintner, 2024, p. 1). Consumers’ AI literacy at the individual level can play a significant moderating role in determining the impact of AI equality (vs. AI divide) on consumers’ responses to, interaction with, and evaluation of AI-driven digital transformation in general, and more specifically, ChatGPT-powered virtual agents in the emerging AI-driven digital marketing and virtual commerce domains. For example, for those consumers scoring higher on AI literacy scales, the positive impact of AI equality on purchase intention can be amplified. In contrast, for those consumers scoring lower on AI literacy scales, the positive impact of AI equality on purchase intention may be minimal whereas the negative impact of the AI divide on purchase intention may be maximal since they may feel incompetent or disempowered to take advantage of the AI infrastructure and resources. Thus, this line of future research may need to further delve into the interaction effects between the micro-psychological-level AI literacy of individuals, meso-organizational-level AI interfaces/infrastructure of companies, and macro-societal-level AI preparedness of the global AI infrastructure/ecosystem.

Fourth, the current study examined AI equality as an antecedent (i.e., independent, exogenous variable) of consumers’ intention to adopt emerging AI technologies, thus implicating the possibility of the “the AI-rich get richer” scenario. The inequitable allocation of capital in AI combined with existing economic gaps can exacerbate wealth inequality (Skare et al., 2024), thus generating a chasm that limits opportunities and further widens the gap between those able to seize, engage with, partake in, harness, and ultimately benefit from the transformative potential of AI technologies (i.e., the AI-rich) and those left on the periphery (i.e., the AI-poor) (Bircan & Özbilgin, 2025). Follow-up studies need to further address this ethical dilemma and tackle the fundamental and philosophical questions of “What is the human race’s ultimate goal in embracing AI-driven digital transformation and pursuing AI equality such as *AI for All*?” and “Does the human race want *AI for All* or not?” Furthermore, measurement of perceived AI equality as the outcome (i.e., dependent, endogenous variable) of a controlled experimental design may further elucidate various cause-and-effect relationships in the ever-evolving, dynamic AI ecosystem. For example, perceived AI equality (vs. AI divide) can be measured as the dependent variable in an experimental design where consumers’ emotions and psychological states are experimentally primed and manipulated as the independent variable in their interaction with AI-enabled agents and virtual influencers in the e-commerce environments.

Fifth, while this research highlights the literature on third-level AI equality and AI-powered virtual influencers as the focal conceptual frameworks, it is important to honestly and truthfully acknowledge the inevitable limitation of the diffused nature of the theoretical discussions. Since the current datasets and empirical findings report the roles of multiple variables including trust (mediator), the need to belong (moderator), privacy concerns (moderator), and perceived benefits of ChatGPT (mediator) in explaining the dynamic relationships between third-level AI equality and behavioral intentions to purchase products promoted by ChatGPT-powered virtual influencers, it is inevitable that a diffused discussion of the various concepts is presented.

Sixth, the participants in this study consist of ChatGPT users recruited only in the United States, thus examining the AI divide (vs. equality) within a single nation/society at the local level. Follow-up studies need to collect data from other regions and cultures, to address the AI divide between the Global North and the Global South at the global level, given that “the economic and social benefits of AI remain geographically concentrated, primarily in the Global North” (World Economic Forum, 2023). AI adoption remains significantly slower in the Global South compared to the Global North (Nugraha, 2025). Therefore, replication of the current conceptual frameworks and methodologies in the Global South context may or may not work, given the contextual caveat in imposing Global North approaches to AI governance and AI sovereignty on the Global South (Laforge, 2024). Future studies among populations from the Global South may need different theoretical frameworks and alternative angles to examine the privacy paradox in the context of the diffusion of ChatGPT as a potential general purpose technology (GPT). The current research is missing the opportunity to engage with the Global South literature on trust in AI, virtual influencers, and ubiquitous ChatGPT–privacy tradeoffs. Comparative insights or contradictions may enrich the emerging discourses about not only the AI divide between the AI-rich and the AI-poor within a specific society, but also the AI divide between the Global North and the Global South. Relatedly, the AI Preparedness Index (AIPI), proposed by the IMF, covers multiple strategic areas for AI readiness: “(1) digital infrastructure, (2) innovation and economic integration, (3) human capital and labor market policies, and (4) regulation and ethics.” (Cazzaniga et al., 2024, p. 20). Future research needs to consider these macro-level dimensions and social factors beyond the micro-level dimensions and psychological factors only narrowly examined in the current study.

## 6. Conclusions

The advent of LLMs not only represents a significant leap forward in the field of artificial intelligence (AI) in general but also marks a significant milestone particularly in the natural language processing (NLP) domain (Cahalane & Kirshner, 2025). NLP plays an integral role in the field of AI since it serves as a bridge between human communication and machine comprehension (Rothman, 2021; Roumeliotis et al., 2024). NLP techniques can empower companies and marketers to derive meaningful insights from vast amounts of unstructured data to facilitate efficient information retrieval, systematic data analyses, and ultimately data-driven decision-making processes (Li et al., 2022; Roumeliotis et al., 2024). The current research is particularly focused on LLM-based artificial agents in AI-driven marketing and e-commerce: *ChatGPT-powered virtual influencers in a human-centered AI ecosystem*. Empirical data and findings from the current research elucidate important factors to consider in Human–AI Interaction (HAII) in the specific domain of AI-powered e-commerce and marketing communication.

Despite several caveats, this research marks a scientific endeavor to address timely and relevant issues in the rapidly evolving AI-powered digital transformation and provides empirical data on AI equality (vs. AI divide), the privacy paradox involving a general purpose technology (GPT), trust in ChatGPT, and social–psychological factors pertinent to ChatGPT-powered virtual influencer marketing in electronic marketplaces. To conclude, the current study addresses ethical dilemmas in developing an equitable AI ecosystem, offers practical implications of the “*privacy paradox*” for designing trustworthy and ubiquitous AI interfaces in the dynamically evolving AI-powered digital transformation landscape and electronic marketplaces, and provides theoretical implications of the ChatGPT epidemic in a humane AI ecosystem for the literature on general purpose technology (GPT).

## Figures and Tables

**Table 1:** List of variables with number of items, results of reliability testing (Cronbach’s alpha), and sample measurement items.

Variable	Number of Items	Cronbach’s Alpha (α)	Sample Items
AI Equality: Third-Level AI Equality (IV)	5	0.830	“Through AI technology, I bought a product more cheaply than I could in the local store” “Through AI technology, I can buy/sell things in a more convenient way” “Through AI technology, I can do business more efficiently”
GPT Trust: Trust in ChatGPT (Mediator)	4	0.782	“ChatGPT is a trustworthy technology” “ChatGPT technology can be relied on to keep its promises” “I can count on ChatGPT technology to protect my privacy”
NTB: Need to Belong (Moderator)	3	0.777	“I do not like being alone” “I have a strong need to belong” “I try hard not to do things that will make other people avoid or reject me”
GPT Ubiquitous: Perceived Benefits of Ubiquitous ChatGPT (Mediator)	3	0.714	“ChatGPT technology can help me be well informed all the time” “ChatGPT technology can allow me to access account information anytime” “ChatGPT technology can help me do transactions regardless of where I am”
GPT Privacy: Privacy Concerns about ChatGPT (Moderator)	3	0.730	“I am concerned that too much personal information is collected when I use ChatGPT-based online shopping websites and apps” “I have doubts about how well my privacy is protected when I use ChatGPT” “I am concerned with the security of sensitive information when I use ChatGPT”
Purchase VI: Intention to Purchase Products Promoted by ChatGPT-Powered Virtual Influencers (DV)	5	0.867	“I am very interested in buying products promoted by ChatGPT-powered virtual influencers in the near future” “I will take ChatGPT-powered virtual influencers into consideration and have them recommend products” “I can imagine purchasing products advertised by ChatGPT-powered virtual influencers in the near future”

**Table 2:** Descriptive statistics and bi-variate Pearson correlation matrix (N = 467).

Variable	*M*	*SD*	1.	2.	3.	4.	5.	6.
1. AI_Equality: Third-Level AI Equality (IV)	5.070	0.944	1					
2. ChatGPT_ Trust: Trust in ChatGPT (Mediator)	5.232	0.891	0.738 **	1				
3. NTB: Need to Belong (Moderator)	4.859	1.196	0.443 **	0.495 **	1			
4. ChatGPT_ Ubiquitous: Perceived Benefits of Ubiquitous ChatGPT (Mediator)	5.238	0.912	0.688 **	0.783 **	0.506 **	1		
5. ChatGPT_ Privacy: Privacy Concerns about ChatGPT (Moderator)	5.077	0.967	0.629 *	0.486 *	0.472 *	0.518 **	1	
6. Intention to Purchase Products Promoted by ChatGPT Virtual Influencers (DV)	5.1126	1.007	0.811 **	0.809 **	0.503 *	0.730 **	0.527 **	1

** *p* < 0.01 (Pearson’s correlation is significant at the 0.01 level, two-tailed). * *p* < 0.05 (Pearson’s correlation is significant at the 0.05 level, two-tailed).

**Table 3:** Mediation analysis (PROCESS Model 4: H3 [top]) and Moderated mediation analysis (PROCESS Model 7: H5 [bottom]).

Direct Relationships (PROCESS Model 4)	Coefficient	*t* Value	*p* Value
Third-Level AI Equality (vs. AI Divide) → Trust in ChatGPT	0.6977	23.7390	0.000
Trust in ChatGPT → Intention to Purchase Products Promoted by ChatGPT-Powered Virtual Influencers	0.5216	13.5185	0.000
Third-Level AI Equality (vs. AI Divide) → Intention to Purchase Products Promoted by ChatGPT-Powered Virtual Influencers	0.4976	13.6814	0.000
**Indirect Relationship (H3)**	Coefficient	Confidence Interval	
		Upper	Lower
Third-Level AI Equality (vs. AI Divide) → Trust in ChatGPT → Intention to Purchase Products Promoted by ChatGPT-Powered Virtual Influencers	0.3639	0.2027	0.5292
**Direct Relationships** **(PROCESS Model 7)**	Coefficient	*t* value	*p* value
Third-Level AI Equality (vs. AI Divide) → Perceived Benefits of Ubiquitous ChatGPT	0.6541	16.3337	0.000
Perceived Benefits of Ubiquitous ChatGPT → Intention to Purchase Products Promoted by ChatGPT-Powered Virtual Influencers	0.3609	9.5510	0.000
Third-Level AI Equality (vs. AI Divide) → Intention to Purchase Products Promoted by ChatGPT-Powered Virtual Influencers	0.6223	17.106	0.000
Third-Level AI Equality (vs. AI Divide) x Privacy Concerns → Perceived Benefits of Ubiquitous ChatGPT	0.1581	7.7333	0.000
**Moderated Mediation (H5)**	Index of Moderated Mediation	Confidence Interval	
		Upper	Lower
Third-Level AI Equality (vs. AI Divide) x Privacy Concerns → Perceived Benefits of Ubiquitous ChatGPT → Intention to Purchase Products Promoted by ChatGPT-Powered Virtual Influencers	0.0570	0.0101	0.1071

## Data Availability

The data presented in this study are available upon request from the corresponding author. (The data are not publicly available due to privacy or ethical restrictions).

## Abbreviations

The following abbreviations are used in this manuscript:

ANN	Artificial Neural Network
LLM	Large Language Model
HAII	Human–AI Interaction
ChatGPT	Chat Generative Pre-Trained Transformer
GPT	General Purpose Technology
GenAI	Generative Artificial Intelligence
